# Supplementary material for: Activation of the Spx redox sensor counters cysteine-driven Fe(II) depletion under disulfide stress
Source: bioRxiv. 2025 Dec 3:2025.12.03.692063. Preprint. [Version 1] doi: 10.64898/2025.12.03.692063 (PMC12822559; doi:10.64898/2025.12.03.692063)
Supplement: Supplementary file 1 [file NIHPP2025.12.03.692063V1-supplement-1.pdf]

bioRxiv preprint doi: https://doi.org/10.64898/2025.12.03.692063; this version posted December 3, 2025. The copyright holder for this preprint (which was not certified by peer review) is the author/funder, who has granted bioRxiv a license to display the preprint in perpetuity. It is made available under a CC-BY 4.0 International license.

Figure 1- figure supplement 1

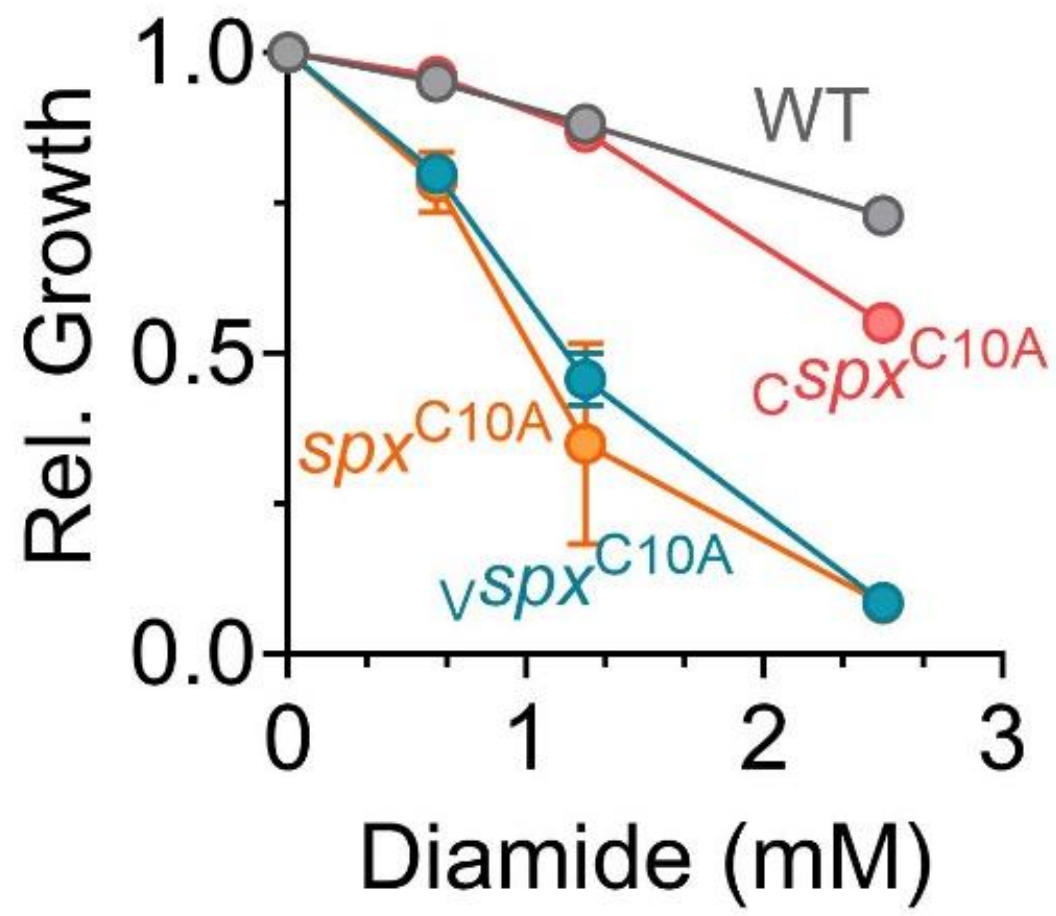

Figure 1- figure supplement 1: Complementation of *spx*^{C10A}. Dose-dependent impact of diamide on growth of various *S. aureus* strains. *$_C$spx*^{C10A}, *spx*^{C10A} mutant complemented with pJC1111: *spx* at SaPI site, *$_V$spx*^{C10A}, *spx*^{C10A} mutant with pJC1111 (vector control) at SaPI site; n= 3, Data represent mean ± SD.

bioRxiv preprint doi: https://doi.org/10.64898/2025.12.03.692063; this version posted December 3, 2025. The copyright holder for this preprint (which was not certified by peer review) is the author/funder, who has granted bioRxiv a license to display the preprint in perpetuity. It is made available under a CC-BY 4.0 International license.

Figure 1- figure supplement 2

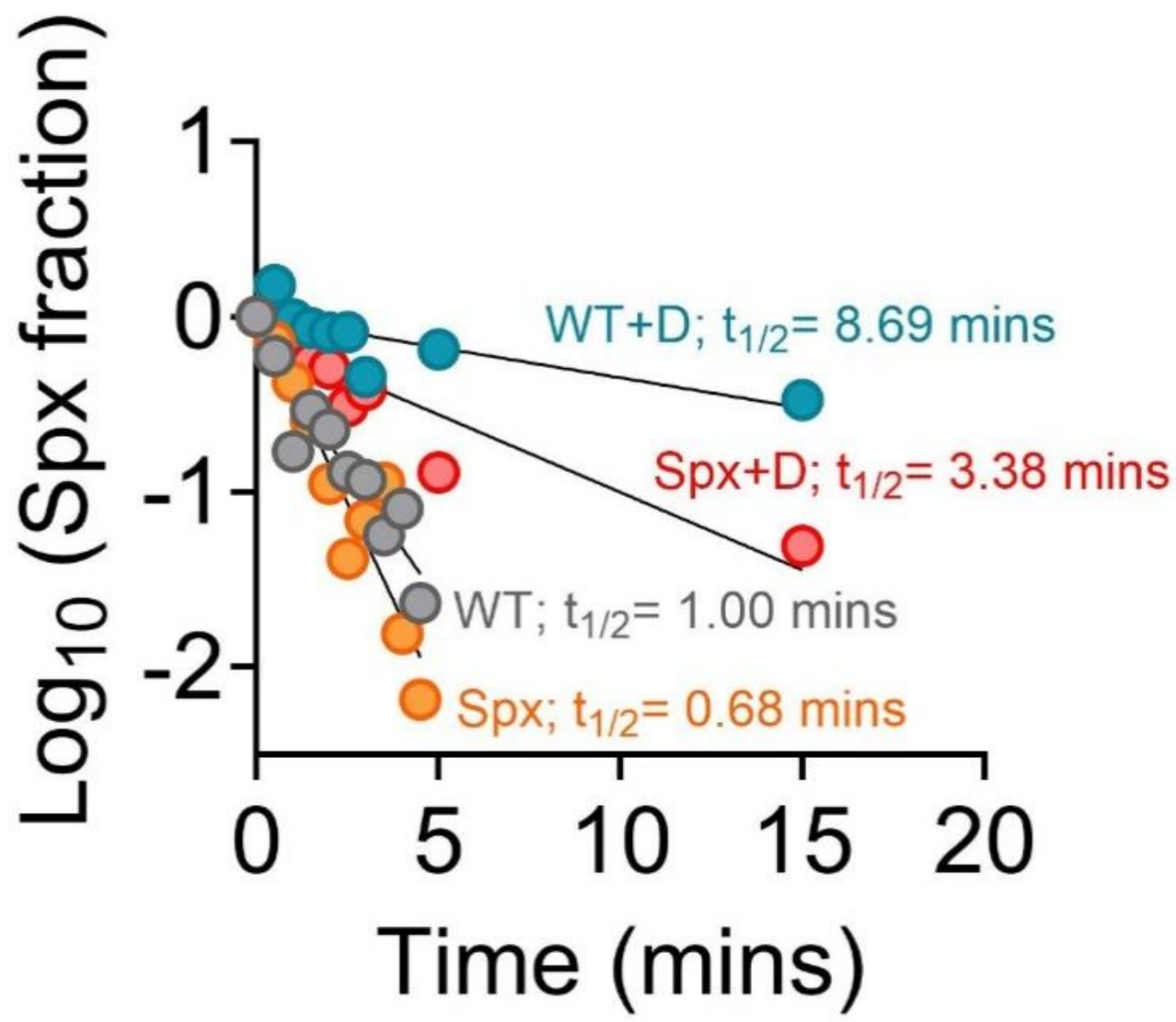

Figure 1- figure supplement 2: Half-life of Spx. The half-life of different Spx variants were determined from Spx decay intensities observed over 0- 15 min for WT+D and *spx*^{C10A}+D, and 0-4.5 min for WT and *spx*^{C10A}. The Spx intensities derived from western blot analysis was normalized to the loading control (enolase). The half-life ($t_{1/2}$) was calculated as the ratio of $Log_{10}(0.5)$ to the slope of the log-transformed protein decay intensity. +D, diamide.

bioRxiv preprint doi: https://doi.org/10.64898/2025.12.03.692063; this version posted December 3, 2025. The copyright holder for this preprint (which was not certified by peer review) is the author/funder, who has granted bioRxiv a license to display the preprint in perpetuity. It is made available under a CC-BY 4.0 International license.

Figure 2- figure supplement 1

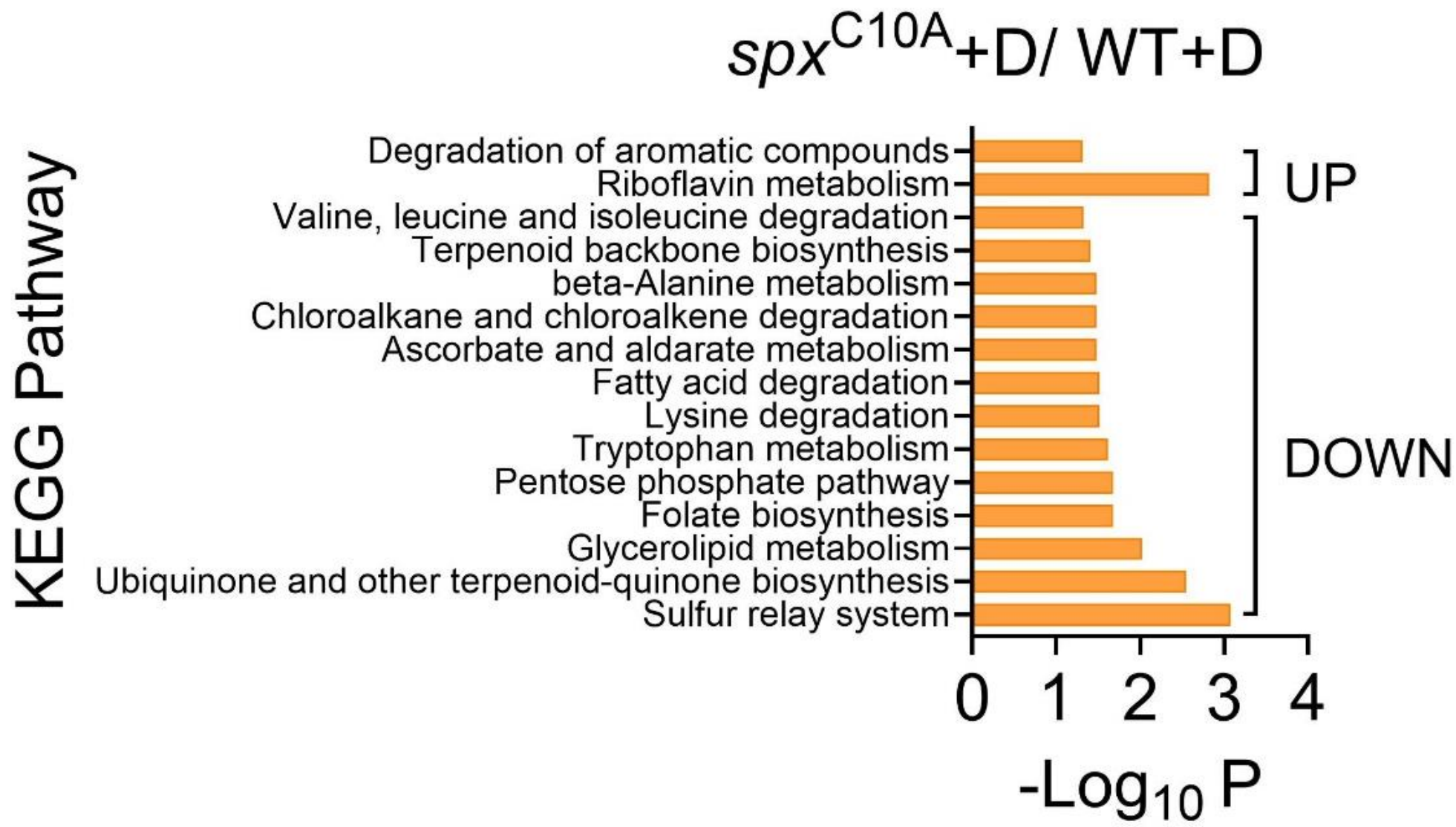

Figure 2- figure supplement 1: KEGG pathway analysis of RNAseq dataset. Differentially expressed pathways in the *spx*^{C10A} mutant relative to the WT strain after 15 mins of 0.5 mM diamide treatment. Analysis was carried out using KOBAS 3.0.

bioRxiv preprint doi: https://doi.org/10.64898/2025.12.03.692063; this version posted December 3, 2025. The copyright holder for this preprint (which was not certified by peer review) is the author/funder, who has granted bioRxiv a license to display the preprint in perpetuity. It is made available under a CC-BY 4.0 International license.

Figure 4- figure supplement 1

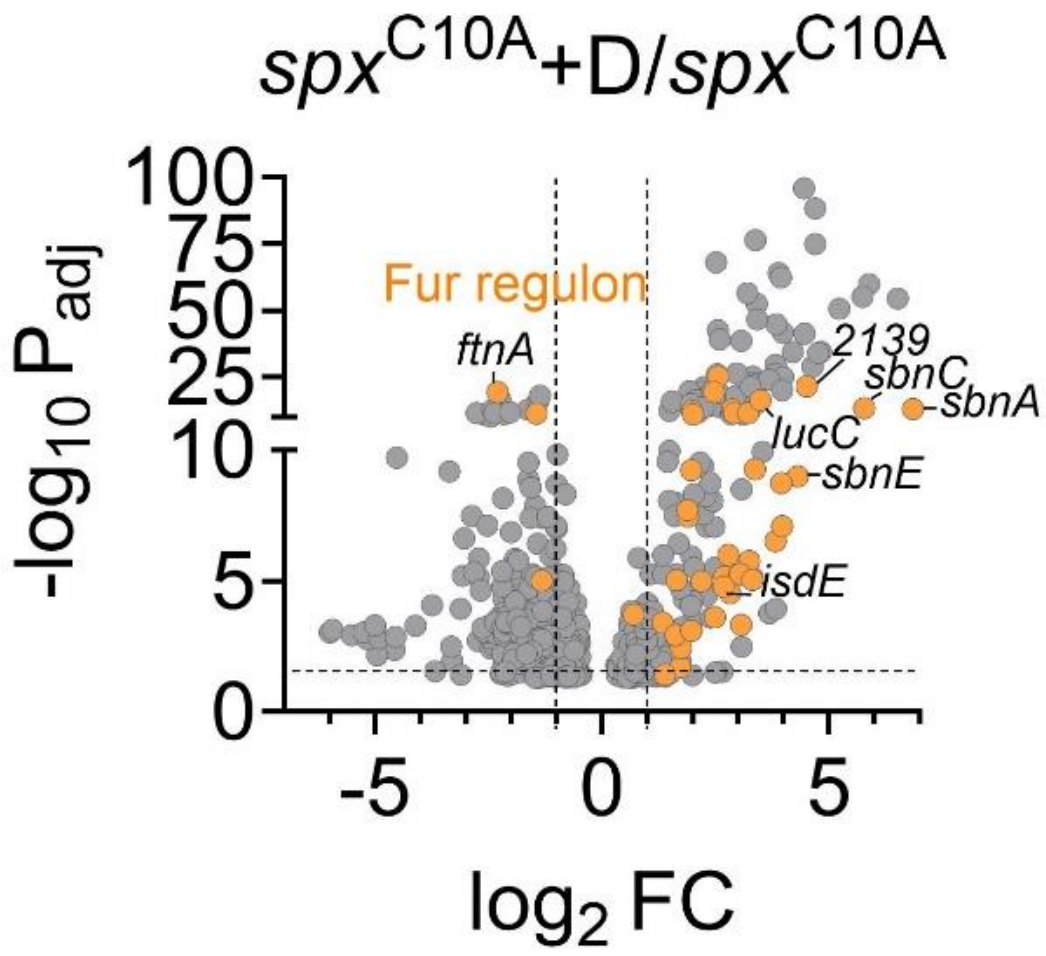

Figure 4- figure supplement 1: Volcano plot. Differentially expressed genes in *spx*^{C10A} mutant strain following diamide-induced disulfide stress. +D, diamide treatment.

bioRxiv preprint doi: https://doi.org/10.64898/2025.12.03.692063; this version posted December 3, 2025. The copyright holder for this preprint (which was not certified by peer review) is the author/funder, who has granted bioRxiv a license to display the preprint in perpetuity. It is made available under a CC-BY 4.0 International license.

Figure 5- figure supplement 1

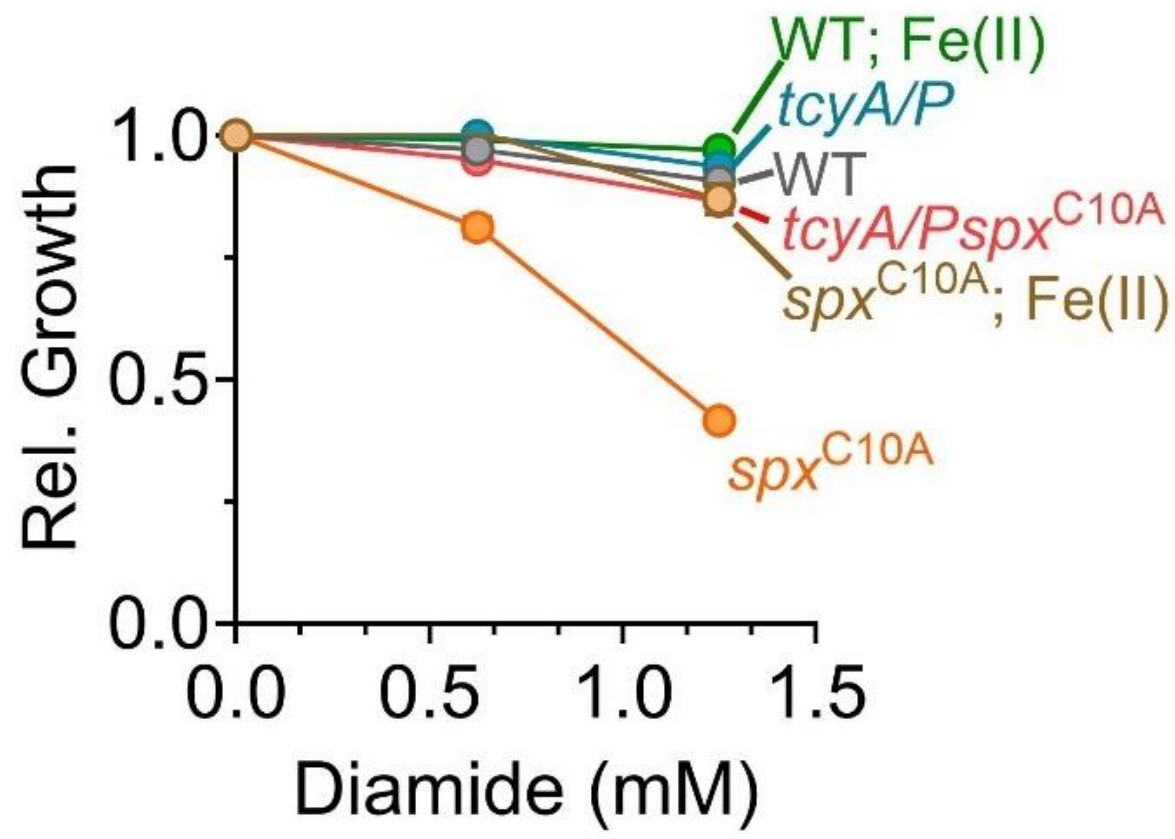

Figure 5- figure supplement 1: L-Cys-mediated Fe(II) starvation in the *spx*^{C10A} mutant under disulfide stress occurs regardless of glucose in the medium. Cultures were grown in TSB (-) glucose with increasing concentrations of diamide. The relative growth was determined from the fractional area under the growth curve of diamide treated cultures to that of untreated culture.

bioRxiv preprint doi: https://doi.org/10.64898/2025.12.03.692063; this version posted December 3, 2025. The copyright holder for this preprint (which was not certified by peer review) is the author/funder, who has granted bioRxiv a license to display the preprint in perpetuity. It is made available under a CC-BY 4.0 International license.

Figure 5- figure supplement 2

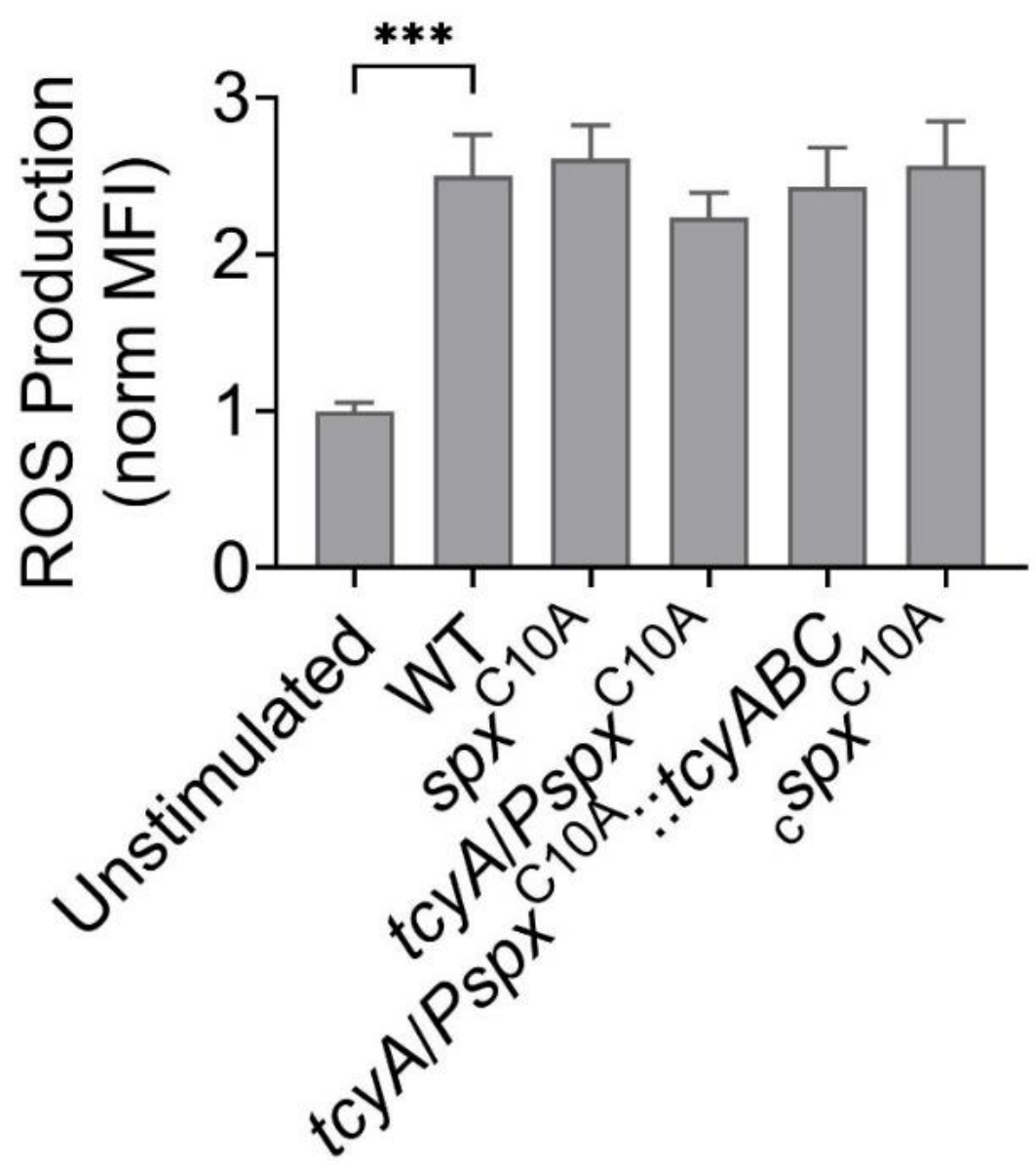

Figure 5- figure supplement 2: ROS levels in neutrophils. Neutrophils were cultured with *S. aureus* (MOI = 10) for two hours. Neutrophils were stained with DHR123, approximately 15 minutes prior to fixation in 4% PFA. Neutrophils were blocked and stained with anti-CD15 and anti-CD16 antibodies and the mean fluorescence intensity (MFI) for DHR123 in the neutrophil population was quantified by flow cytometry. MFI was normalized to unstimulated neutrophils. n= 5, mean ± SEM, One-way ANOVA, Tukey's post-test, ****P* ≤ 0.001.

bioRxiv preprint doi: https://doi.org/10.64898/2025.12.03.692063; this version posted December 3, 2025. The copyright holder for this preprint (which was not certified by peer review) is the author/funder, who has granted bioRxiv a license to display the preprint in perpetuity. It is made available under a CC-BY 4.0 International license.

Supplemental Table 1: Strains and plasmids used in this study

Strain	Description	Source
E. coli Electro10 Blue	*E. coli* strain used for plasmid maintenance	Agilent Tech.
S. aureus JE2	*S. aureus* USA300 LAC 13C cured of endogenous plasmids	(53)
RN4220	Restriction deficient strain used as a transformation intermediate	(54)
spx^{C10A}	*spx* Cys10→ Ala substitution in JE2	(13)
spx^{C13A}	*spx* Cys13→ Ala substitution in JE2	(13)
spx^{C10,13A}	*spx* Cys10,13→ Ala substitution in JE2	(13)
$_{C}$*spx*^{C10A}	*spx*^{C10A} mutant complemented with native *spx* allele in *S. aureus* SaPI site using pJC1111	(13)
$_{V}$*spx*^{C10A}	*spx*^{C10A} mutant containing the pJC1111 integrated in *S. aureus* SaPI site	(13)
clpP	*Bursa aurealis* transposon mutant, Erm^R	(5)
spx^{C10A} *clpP*	*clpP*::Tn Erm^R mutation in *spx*^{C10A}	This study
tcyP	*Bursa aurealis* transposon mutant, Erm^R	(53)
tcyA	*Bursa aurealis* transposon mutant, Kan^R	This study
tcyA/*P*	*tcyA*::Tn Kan^R, *tcyP*::Tn Erm^R	This study
spx^{C10A} *tcyA*	*tcyA::*Tn Kan^R mutation in *spx*^{C10A}	This study
tcyA/*P* :: *tcyABC*	*tcyA*/*P* mutant complemented with *tcyABC* in SaPI site	This study
spx^{C10A} *tcyA*/*P*	*tcyA*::Tn Kan^R, *tcyP*::Tn Erm^R in *spx*^{C10A}	This study
spx^{C10A} *tcyA*/*P* :: *tcyABC*	*spx*^{C10A} *tcyA*/*P* mutant complemented with *tcyABC* in SaPI site	This study
fur	*Bursa aurealis* transposon mutant, Erm^R	(53)
fur spx^{C10A}	*fur*::Tn Erm^R mutation *in spx*^{C10A}	This study
**<u>Plasmid</u>**		
pJC1111	SaPI integration vector	(48)
pVT4	Brx-roGFP2 under control of constitutive P_1*sarA*	This study
pAH6.5	pJC1111:: *tcyABC*	This study
pAH5.5	pJC1111:: *fur*	This study

bioRxiv preprint doi: https://doi.org/10.64898/2025.12.03.692063; this version posted December 3, 2025. The copyright holder for this preprint (which was not certified by peer review) is the author/funder, who has granted bioRxiv a license to display the preprint in perpetuity. It is made available under a CC-BY 4.0 International license.

Supplemental Table 2: Primers used in this study

Gene/ modification	Primer name	Primer sequence 5'-3'
tcyABC complementation	pAH6.5_tcyABC_fwd	GAGCCGCTGCATGCCTGCAGATATAAGTTGCTAGCTATATAAAGC
	pAH6.5_tcyABC_rev	AGCTCGGTACCCGGGGATCCAACATAACAAAGAACCACG
fur complementation	pAH5.5_fur_fwd	CGGCCGCTGCATGCCTGCAGCAAATGAGAAAAGCTTGC
	pAH5.5_fur_rev	AGCTCGGTACCCGGGGATCCAGCGAAGGTTAATTTAACC
